# INTENSIVE CARE UNIT PRESCRIPTIONS MUST FIT RISK FACTORS TO PREVENT STRESS ULCER BLEEDING

**DOI:** 10.1590/0102-672020210003e1587

**Published:** 2022-01-05

**Authors:** Rodolfo Castro Cesar de OLIVEIRA, Osvaldo MALAFAIA, Fernando Issamu TABUSHI, Carlos Roberto NAUFEL, Elora Sampaio LOURENCO, Felipe Yoshio TABUSHI

**Affiliations:** 1 Mackenzie Evangelical Faculty of Paraná, Curitiba, PR, Brazil; 2 University Evangelical Mackenzie Hospital, Curitiba, PR, Brazil

**Keywords:** Stress, physiological, Hemorrhage, Peptic ulcer, Critical care, Primary prevention, Estresse fisiológico, Hemorragia, Úlcera péptica, Cuidados críticos, Prevenção de doenças

## Abstract

***Background*:**

The physiological stress of critically ill patients can trigger several complications, including digestive bleeding due to stress ulcers (DBSU). The use of acid secretion suppressants to reduce their incidence has become widely used, but with the current understanding of the risks of these drugs, their use, as prophylaxis in critically ill patients, is limited to the patients with established risk factors.

**Aim:**

To determine the appropriateness of the use of prophylaxis for stress ulcer bleeding in acutely ill patients admitted to intensive care units and to analyze the association of risk factors with adherence to the prophylaxis guideline.

**Methods:**

Retrospective, analytical study carried out in three general adult intensive care units. Electronic medical records were analyzed for epidemiological data, risk factors for DBSU, use of stress ulcer prophylaxis, occurrence of any digestive bleeding and confirmed DBSU. The daily analysis of risk factors and prophylaxis use were in accordance with criteria based on the Guidelines of the Portuguese Society of Intensive Care for stress ulcer prophylaxis.

**Results:**

One hundred and five patients were included. Of the patient days with the opportunity to prescribe prophylaxis, compliance was observed in 95.1%. Of the prescription days, 82.35% were considered to be of appropriate use. Overt digestive bleeding occurred in 3.81% of those included. The occurrence of confirmed DBSU was identified at 0.95%. Multivariate analysis by logistic regression did not identify risk factors independently associated with adherence to the guideline, but identified risk factors with a negative association, which were spinal cord injury (OR 0.02 p <0.01) and shock (OR 0.36 p=0.024).

**Conclusion:**

The present study showed a high rate of adherence to stress ulcer prophylaxis, but with inappropriate use still significant. In the indication of prophylaxis, attention should be paid to patients with spinal cord injury and in shock.

## RESUMO

**Racional:**

O estresse fisiológico dos pacientes críticos pode desencadear várias complicações, entre elas o sangramento digestivo por úlcera de estresse (SDUE). O uso de supressores da secreção ácida para reduzir sua incidência passou a ser amplamente utilizado, mas com o atual entendimento dos riscos destes medicamentos sua utilização, como profilaxia em doentes críticos, está limitada aos pacientes com fatores de risco estabelecidos.

**Objetivos:**

Determinar a adequação do uso de profilaxia para sangramento por úlcera de estresse em pacientes agudamente enfermos internados em unidades de terapia intensiva e analisar a associação dos fatores de risco com a adesão à diretriz de profilaxia.

**Métodos:**

Estudo retrospectivo, analítico, realizado em três unidades de terapia intensiva gerais de adultos. Os prontuários eletrônicos foram analisados para dados epidemiológicos, fatores de risco para SDUE, uso de profilaxia para SDUE, ocorrência de qualquer sangramento digestivo e de SDUE confirmado. A análise diária dos fatores de risco e uso de profilaxia foram de acordo com critérios baseados nas Diretrizes da Sociedade Portuguesa de Cuidados Intensivos para profilaxia da úlcera de estresse.

**Resultados:**

foram incluídos 105 pacientes. Dos pacientes-dia com oportunidade de prescrição de profilaxia, foi observada adesão em 95,1%. Dos dias de prescrição foram considerados de uso apropriado 82,35%. Sangramento digestivo visível ocorreu em 3,81% dos incluídos. A ocorrência de SDUE confirmado foi identificada em 0,95%. A análise multivariada por regressão logística não identificou fatores de risco independentemente associados com a adesão à diretriz, mas identificou fatores de risco com associação negativa, que foram lesão da medula espinhal (OR 0.02 p<0,01) e choque (OR 0.36 p=0.024).

**Conclusão:**

O presente estudo evidenciou alta taxa de adesão à profilaxia para SDUE, mas com uso inapropriado ainda significativo. Na indicação de profilaxia deve-se ter atenção aos pacientes com lesão de medula espinhal e choque.

## INTRODUCTION

In 1935 Hans Selye, based on experimentation on rats, observed what he called “general adaptation syndrome”, which occurred when the organism suffered acute and severe damage by a non-specific agent and which, in its first stage (6-48 h), one of the manifestations was the formation of acute erosions of the digestive tract8. Later the author started to use the term stress, as in his monograph “The Physiology and Pathology of Exposure to Stress” from 1950.

In critically ill patients, factors that impair the integrity of the gastric mucosa are present1. As a result, acute gastric mucosal damage (stress ulcer) or bleeding from stress ulcer may occur. With the observation of this occurrence, the practice of using pharmacological agents for the prevention of gastrointestinal bleeding for stress ulcer (GIBSU) in critical patients has become widespread. Such use has risks and, therefore, should be indicated in patients with an adequate risk-benefit profile. Currently, in critically ill patients hospitalized for acute illness, only those most at risk for GIBSU have an indication for pharmacoprophylaxis^10^. Even so, studies show that inappropriate use still occurs frequently^7^.

The aim of this study was to determine the appropriateness of the use of prophylaxis for GIBSU in acutely ill patients admitted to intensive care units (ICU) and to analyze the association of risk factors with adherence to prophylaxis for GIBSU.

## METHODS

This is a retrospective, analytical research, carried out in three general ICUs of the University Evangelical Mackenzie Hospital, Curitiba, PR, Brazil, with adult. The sample consisted of all patients discharged or died, from the three ICUs between January and February 2020. Inclusion criteria were: age over 18 years and hospitalization with discharge within the established period. The exclusion criteria were: age below 18 years; patients with convincing indication of non-prophylactic acid suppression upon admission; and those electively admitted to the ICUs after procedures.

### Data collect

Electronic medical records were analyzed to obtain the following data in relation to GIBSU: 1) gender; 2) age; 3) data on admission to the ICU; 4) inpatient sector; 5) diagnosis of hospitalization; 6) comorbidities, degree of dependence on the Fungulin scale, Charlson’s comorbidity index; 7) presence of risk factors; 8) prescription of prophylactic medication; 9) medication used for GIBSU prophylaxis (drug, dose, route and frequency); 10) visible digestive bleeding and endoscopically confirming GIBSU in the ICU; 11) length of stay; and 12) type of exit from the ICU (death or transfer).

Data on risk factors and prophylaxis use for GIBSU were recorded individually for each day of hospitalization. Day patients were no longer registered in cases where they evolved to indicate non-prophylactic acid suppression or for palliation (where prophylaxis for GIBSU could be considered therapeutic futility).

Indications for the use of pharmacological prophylaxis for GIBSU were based on the guidelines of the Portuguese Society of Intensive Care for Stress Ulcer Prophylaxis in the Intensive Care Unit. Prescription of proton pump inhibitor by enteral or parenteral route, H2 antagonists by enteral or parenteral route was considered prophylaxis for GIBSU in the absence of therapeutic indication.

### Statistical analysis

Was performed using the Chi-square test and Fisher’s test to compare categorical variables. Multivariate analysis with logistic regression of risk factors with p less than or equal to 0.1. 95% confidence intervals were adopted. For the analysis, the software Epi Info v 7.2 (https://www.cdc.gov/epiinfo/index.html) was used.

## RESULTS

One hundred and seventy-three patients left the ICUs. Of these, 68 were excluded, 32 were indicated for non-prophylactic acid suppression (Table 1), 35 for elective admission to the ICU after therapeutic procedures and one under 18 years of age on admission.

**TABLE 1:** Convincing indications for the use of H2 antagonists or PPIs of patients excluded from the study

Indication	n	% of total excluded
Suspected or confirmed upper gastrointestinal bleeding	13	37.14%
Dual antiplatelet therapy or therapeutic anticoagulant use	11	31.43%
Chronic use of PPI or H2 antagonist	5	14.29%
Known peptic disease in the healing and maintenance phase	3	8.57%
GERD and complications associated with acidity	3	8.57%

After the exclusions, 105 subjects were included. Some characteristics of the sample are described in Table 2.

**TABLE 2:** Characteristics of the studied population

Characteristic	% or average (min-max, median)	Standard deviation
Age (years)	50.38 (18-87, 51)	18.74
Male gender	77.14%	
ICU stay (days)	8.71 (0-62, 5)	10.05
Death in the ICU	46.67%	
Surgical	67.62%	
Fungulin score at the entrance	32.51 (11-42, 34)	5.76
Charlson’s comorbidity index	2.27 (0-9, 2)	2.51

The most common entry diagnoses were: multiple trauma (n=17, 16.19%), burn (n=13, 12.38%), sepsis (n=13, 12.38%), traumatic brain injury (n=9, 8.57%) and hemorrhagic stroke (n=7, 6.67%, Table 3).

The most frequent comorbidities were systemic arterial hypertension (24.76%), diabetes mellitus (13.33%), alcoholism (10.48%) and chronic obstructive pulmonary disease (7.62%, Table 3).

Four occurrences of visible digestive bleeding were identified (3.81%, 95% CI 1.05-9.47). Two of these patients underwent upper digestive endoscopy, one of whom was diagnosed with esophagogastroduodenal ischemia and the other with stress ulcers, which represents a 0.95% incidence of bleeding confirmed by stress ulcers (95% CI 0.02-5.19).

Eight hundred and three patient-days were analyzed. Of these, 633 were identified with the opportunity to prescribe prophylaxis for GIBSU with 95.1% adherence (95% CI 93.13-96.53). There were 731 patient-days with prescription of prophylaxis for it. Of these, PPIs were prescribed in 56.77%, in 42.82% H2 antagonists and in 0.41% PPI and H2 antagonist association (Table 4).

**TABLE 3:** Diagnosis and comorbidities at the entrance

Diagnosis	n	%
Polytrauma	17	16.19%
Burn	13	12.38%
Sepsis	13	12.38%
Traumatic brain injury	9	8.57%
Hemorrhagic stroke	7	6.67%
Pneumonia	4	3.81%
Acute abdomen	3	2.86%
Ischemic stroke	3	2.86%
Seizure	3	2.86%
Status epilepticus	3	2.86%
Respiratory failure	3	2.86%
Acute pulmonary edema	2	1.9%
White weapon injury	2	1.9%
Acute renal failure	2	1.9%
Neoplasm of the central nervous system	2	1.9%
Retropharyngeal abscess	1	0.95%
Abscess of the central nervous system	1	0.95%
Shock	1	0.95%
Cholangitis	1	0.95%
Coma	1	0.95%
Firearm injury	1	0.95%
Fournier syndrome	1	0.95%
Subdural hematoma	1	0.95%
Hemoptysis	1	0.95%
Subarachnoid hemorrhage	1	0.95%
Normobaric hydrocephalus	1	0.95%
Arterial hypertension	1	0.95%
Nephrolithiasis	1	0.95%
Acute arterial occlusion	1	0.95%
Pancreatitis	1	0.95%
Diabetic foot	1	0.95%
Pneumothorax	1	0.95%
Bladder bleeding after cancer surgery	1	0.95%
Face trauma	1	0.95%
Total	105	100%
Comorbidity	n	%
Systemic arterial hypertension	26	24.76%
Diabetes mellitus	14	13.33%
Alcoholism	11	10.48%
Chronic obstructive pulmonary disease	8	7.62%
Stroke	6	5.71%
Dyslipidemia	6	5.71%
Epilepsy	6	5.71%
Chronic kidney failure	6	5.71%
Hypothyroidism	5	4.76%
Metastatic neoplasia	5	4.76%
Coronary artery disease	4	3.81%
Obesity	4	3.81%
Drug addiction	3	2.86%
Human immunodeficiency virus infection	3	2.86%
Nephrolithiasis	3	2.86%
Smoking	3	2.86%
Asthma	2	1.90%
Chagas disease	2	1.90%
Dementia	1	0.95%
Depression	1	0.95%
Peripheral obstructive arterial disease	1	0.95%
Parkinson’s disease	1	0.95%
Spondylitis	1	0.95%
Schizophrenia	1	0.95%
Atrial fibrillation	1	0.95%
Spine fracture	1	0.95%
Chronic viral hepatitis	1	0.95%
Previous acute myocardial infarction	1	0.95%
Congestive heart failure	1	0.95%
Leukemia	1	0.95%
Lymphoma	1	0.95%
Cerebral palsy	1	0.95%
Klinefelter syndrome	1	0.95%
Peripheral venous thrombosis	1	0.95%
Extrapulmonary tuberculosis	1	0.95%

**TABLE 4:** Doses of drugs prescribed for GBSU prophylaxis

Medication	Dose, route and schedule	Prescription days
Pantoprazole	40 mg IV q24hr	382
Pantoprazole	40 mg IV q12hr	7
Ranitidine	50 mg IV q8hr	156
Ranitidine	150 mg SG q12hr	29
Ranitidine	50 mg IV q12hr	24
Ranitidine	150 mg VO q12hr	10
Ranitidine	50 mg IV q24hr	2
Cimetidine	300 mg IV q8hr	73
Cimetidine	300 mg IV q12hr	13
Cimetidine	300 mg IV q6hr	6
Omeprazole	20 mg VO q24hr	24
Omeprazole	20 mg SG q24hr	1
Omeprazole	40 mg VO q24hr	1
Pantoprazole + ranitidine	40 mg IV q24hr + 50 mg IV q8hr	3

Of the days of prophylaxis prescription, 82.35% (95% CI 79.42-84.95) were considered appropriate and the others inappropriate.

The analysis of the association of risk factors for GIBSU with adherence to prophylaxis in patients with indication is shown in Table 5.

**TABLE 5:** Analysis of the association of risk factors for GIBSU and adherence to prophylaxis

Risk factor	OR	p
Mechanical ventilation	1.48	0.392
Significant coagulopathy	0.36	0.056
Sepsis	1.36	0.437
Burn >35% total body surface	3.95	0.042
Spinal cord injury	0.06	0.003
Cranioencephalic trauma with Glasgow coma scale less than 9	0.39	0.026
Acute or chronic renal failure with renal replacement therapy	0.52	0.203
Corticosteroid use >hydrocortisone 250 mg daily or equivalent	0.71	0.571
Polytrauma with trauma severity index 16 points or more	0.47	0.053
Shock	0.51	0.079

The multivariate analysis of the association of risk factors for GIBSU showed a negative association of spinal cord injury (OR=0.02, p<0.001) and shock (OR=0.36, p=0.024) with adherence to GIBSU prophylaxis. Burn >35% and cranioencephalic trauma resulted in OR=3.03, p 0.144 and OR=0.18, p=0.06, respectively.

## DISCUSSION

The population analyzed was adult patients, acutely ill hospitalized in the ICU. From the analysis of the epidemiological data it can be seen that the patients were predominantly surgical and men, with an average age of 50 years. Considering that the median of the Fungulin score was 34, it is understood that the majority had a degree of dependence compatible with intensive and semi-intensive care^3^.

Regarding the occurrence of digestive bleeding, there was a record of four visible bleeds, that is, 3.81% (95% CI 1.05-9.47), and in only two cases did upper gastrointestinal endoscopy be performed, and a confirmed case of digestive bleeding from stress ulcers (0.95%).

Considering the retrospective methodology of the research and based only on the electronic medical record, it cannot be said that only these cases of digestive bleeding are treated, as bleedings may have occurred and not have been properly registered. Diagnostic confirmation of the bleeding etiology may present difficulties in the population of critically ill patients, in view of the risk of displacement and complications during the transfer between the ICU and the diagnostic sector. Studies that were based on endoscopic evaluation found higher data than in the present study, such as Kumar & Sudhakar^4^ that identified 4.35% of clinically evident GIBSU.

Like all medication, drug prophylaxis for GIBSU should be indicated considering the risk-benefit profile. The inappropriate prescription has several potential problems, such as the risk of adverse events related to the drugs used (and with them the damage to patients, cost of treatment of adverse events and bed occupation for this treatment), as well as the cost of these medications which, although it is not individually high, considering the frequency of use, it can add a significant amount to the institution and the health system.

Considering that the medications of choice for the prophylaxis of GIBSU in the most recent guidelines are PPIs, the predominance of their use, especially pantoprazole, is expected^5^ ^,^ ^10^. It was observed that the predominance of the use of PPIs was also maintained in the subgroup of prescriptions considered inappropriate.

Several different dosages per drug were observed. Some can be explained in view of the patients’ own conditions, such as dose adjustments of H2 antagonists in case of renal failure. There are more widely known risk factors. The analysis of the association of risk factors for GIBSU with adherence to prophylaxis identified burns >35% of the body surface as a factor associated with adherence, but this association was not maintained after the multivariate analysis. Burned patients were identified from the beginning of studies with GIBSU as being at risk, so much so that in their study published in 1994, Cook et al^2^ sought to identify risk factors and excluded the recommendation not to use prophylaxis for GIBSU in patients with burns >30%.

With the multivariate analysis of risk factors, the presence of spinal cord injury and shock demonstrated an independent and negative association with adherence to the prescription of prophylaxis of GIBSU. Santos et al.^7^ observed in their study an association of adherence with mechanical ventilation and coagulopathy, and negative with anticoagulation. In the present study, patients on anticoagulation were excluded because it was understood that their use would be a convincing indication for the use of H2 antagonists or PPIs.

## CONCLUSION

The present study showed a high rate of adherence to prophylaxis for GIBSU, but with inappropriate use still significant. In the indication of prophylaxis, special attention should be paid to patients with spinal cord injury and shock.
